# Stigmasterol alleviates endplate chondrocyte degeneration through inducing mitophagy by enhancing PINK1 mRNA acetylation via the ESR1/NAT10 axis

**DOI:** 10.1515/biol-2022-0913

**Published:** 2025-04-08

**Authors:** Hao Li, Xiaofeng Chen, Baoci Huang, Junjie He, Junxian Xie, Weijun Guo, Jinjun Liang, Jiajian Ruan, Jincheng Liu, Zhen Xiang, Lixin Zhu

**Affiliations:** Department of Spinal Surgery, Orthopedic Medical Center, Zhujiang Hospital, Southern Medical University, Guangzhou 510280, China; Department of Orthopedics, Panyu Hospital of Chinese Medicine, Guangzhou, Guangdong 511400, China; Department of Ultrasound, Guangdong Second Provincial General Hospital, Guangzhou, Guangdong 510310, China

**Keywords:** intervertebral disc degeneration, endplate chondrocyte degeneration, stigmasterol, mitophagy, RNA acetylation

## Abstract

Intervertebral disc degeneration (IVDD) is a core factor in spinal degeneration. To date, there is no effective treatment for IVDD. It is urgent to identify the pathogenesis of IVDD to develop effective strategies for IVDD treatment. Alleviating endplate chondrocyte degeneration is a promising strategy for IVDD treatment, while mitophagy prevents degeneration of endplate chondrocytes. Stigmasterol (STM) protects neurons from injuries by triggering mitophagy, yet the effect of STM on the mitophagy of endplate chondrocytes in IVDD has not been reported. In this study, endplate chondrocyte degeneration was induced by interleukin-1β, and the ribonucleic acid (RNA) acetylation level was identified by acetylated RNA immunoprecipitation. Herein, results indicated that STM alleviated endplate chondrocyte degeneration. Besides, STM induced PTEN-induced kinase 1 (PINK1)-mediated mitophagy in degenerated endplate chondrocytes. Moreover, *N*‐acetyltransferase 10 (NAT10) increased PINK1 expression by improving PINK1 mRNA acetylation in endplate chondrocytes. In addition, STM regulated NAT10 expression by estrogen receptor 1 (ESR1) in degenerated endplate chondrocytes. In summary, the present study revealed that STM attenuated endplate chondrocyte degeneration through inducing mitophagy by enhancing PINK1 mRNA acetylation via the ESR1/NAT10 axis. These findings would provide novel strategies for the treatment of IVDD.

## 1 Introduction

Intervertebral disc degeneration (IVDD) is a core factor in spinal degeneration, leading to intervertebral disc herniation, low back pain, and disability in severe cases [1,2]. Nearly 80% of adults worldwide are affected by IVDD, especially the elderly [3]. IVDD patients suffer from physical and mental health burdens, resulting in a serious decline in life quality [4]. To date, the standard treatments for IVDD include drug therapy, physical therapy, spinal fusion, or joint replacement surgery. However, the low success rate or high cost limits the effectiveness of the above treatments [5]. Therefore, it is urgent to identify the pathogenesis of IVDD to explore new therapeutic targets and develop more effective strategies for IVDD.

The intervertebral disc is mainly composed of nucleus pulposus, annulus fibrosus, and cartilage endplate [6]. As the main source of extracellular matrix, endplate chondrocytes play an important role in the physical buffering capacity and nutritional maintenance of the intervertebral disc [6,7]. Besides, endplate degeneration caused by degeneration of endplate chondrocytes is an important cause of IVDD [8,9]. Thus, alleviating the degeneration of endplate chondrocytes is a promising strategy for IVDD treatment.

Mitochondrial dysfunction contributes to IVDD [10,11], while mitophagy, a specific form of autophagy, could attenuate IVDD by suppressing the degeneration of endplate chondrocytes via preventing mitochondrial dysfunction. For instance, icariin ameliorates IVDD by suppressing the degeneration of endplate chondrocytes and by inducing mitophagy [12]. By contrast, intermittent cyclic mechanical compression promotes IVDD by enhancing endplate chondrocyte degeneration via hindering PTEN-induced kinase 1 (PINK1)-mediated mitophagy [13]. Therefore, therapeutic inducement of mitophagy could prevent the degeneration of endplate chondrocytes to alleviate IVDD.

Stigmasterol (STM), one of the major active ingredients of *Achyranthes bidentata*, has been shown to protect against hypoxia/reoxygenation-induced injuries by triggering mitophagy in hippocampal neurons [14]. Similarly, STM ameliorates glutamate-induced neurotoxicity by promoting mitophagy of HT-22 cells [15]. However, the effect of STM on mitophagy of endplate chondrocytes in IVDD has not been reported.

*N*‐acetyltransferase 10 (NAT10)-mediated *N*4‐acetylcytidine (ac4C) acetylation is a highly conserved modification of ribonucleic acid (RNA) by improving RNA stability [16]. Therefore, mRNA acetylation plays a critical role in development, cancer, premature disease, and viral infection [17]. Nevertheless, the effect of RNA acetylation on IVDD or degeneration of endplate chondrocytes is largely unknown.

Therefore, the primary aim of this study was to investigate whether STM could prevent the degeneration of endplate chondrocytes by restoring mitophagy via modifying RNA acetylation in IVDD.

## 2 Materials and methods

### 2.1 Cell culture

Primary mouse endplate chondrocytes were purchased from Hechuang Biotech (Guangzhou, Guangdong, China). Then, cells were cultured with Chondrocyte Medium (#4651, ScienCell, Carlsbad, CA, USA) containing 10% fetal bovine serum (#SH30087.01, HyClone, Logan, UT, USA) and 100 U/mL penicillin–streptomycin (#SH30010, HyClone) in a humidified atmosphere of 5% CO_2_ at 37°C.

### 2.2 Cell treatment

To induce degeneration, mouse endplate chondrocytes were treated with 10 ng/mL interleukin-1β (IL-1β) (#bs-41073P, Bioss, Beijing, China) for 24 h. Besides, mouse endplate chondrocytes were treated with 5, 10, and 15 μg/mL STM diluted in DMSO (#IS0760, Solarbio, Beijing, China) for 48 h after IL-1β treatment to determine the effect of STM on endplate chondrocyte degeneration. Moreover, small interfering RNA (siRNA) targeting PINK1, NAT10, and estrogen receptor 1 (ESR1) were transfected into endplate chondrocytes using Lipofectamine 2000 (#11668019, Invitrogen, Carlsbad, CA, USA). The siRNA sequences are listed in Table 1.

**Table 1:** Sequences of siRNAs

Genes		Sequences (5′−3′)
PINK1 siRNA	Sense	UUUUCUGUGUAAAAAUUGCCU
	Antisense	GCAAUUUUUACACAGAAAACC
NAT10 siRNA	Sense	AGUACAAGCGGGAAAUGGCUG
	Antisense	GCCAUUUCCCGCUUGUACUUC
ESR1 siRNA	Sense	AUUUCAUGUUGUAGAGAUGCU
	Antisense	CAUCUCUACAACAUGAAAUGC
siRNA NC	Sense	UUCUCCGAACGUGUCACGU
	Antisense	ACGUGACACGUUCGGAGAA

### 2.3 Bioinformatics analysis

A network was constructed by Cytoscape based on the HERB database (herb.ac.cn/) to analyze the correlation between STM and genes involved in mitophagy. Besides, ac4C sites in PINK1 mRNA were predicted using PACES (www.rnanut.net/paces/). Moreover, the binding sites of ESR1 in the *NAT10* gene promoter were analyzed by the JASPAR database (jaspar.genereg.net/).

### 2.4 Real-time reverse transcription-PCR (qRT-PCR)

Total RNA from mouse endplate chondrocytes was extracted using TRIZOL reagent (#15596026, Invitrogen) followed by the synthesis of cDNA via reverse transcription by PrimeScript II 1st Strand cDNA Synthesis Kit (#6210A, Takara, Dalian, Liaoning, China). Then, qRT-PCR was performed by TB Green^®^ Premix Ex Taq™ (Tli RNaseH Plus) (#RR420A, Takara), and the amount of target RNA was normalized to that of β-actin (internal control). Subsequently, data of qRT-PCR were given by 2^−△△Ct^ compared to that of the control group. The primers used for qRT-PCR are listed in Table 2.

**Table 2:** Sequences of primers

Genes		Sequences (5′−3′)	Amplicon length (bp)
MMP3	Forward	TGGCATTCAGTCCCTCTATGG	198
	Reverse	AGGACAAAGCAGGATCACAGTT	
MMP13	Forward	CTTTTCCTCCTGGACCAAACT	105
	Reverse	TCATGGGCAGCAACAATAAA	
PINK1	Forward	TCGCACACTGTTCCTCGTTA	204
	Reverse	CAGGGACAGCCATCTGAGTC	
NAT10	Forward	AGATGAAGCTGCCGCTATTC	193
	Reverse	TGTCGTTGTGGTCTTGTTCTC	
ESR1	Forward	GCCTAGCTCAGCTCCTTCTCA	109
	Reverse	AGGTCATAGAGGGGCACAACG	
β-actin	Forward	TGTGTCCGTCGTGGATCTGA	150
	Reverse	TTGCTGTTGAAGTCGCAGGAG	

### 2.5 Immunofluorescence (IF)

First, mouse endplate chondrocytes plated on the slide were incubated with 100 nM MitoTracker Red (MTR, #M7512, Invitrogen) for 30 min at 37°C. Then, endplate chondrocytes were fixed by 4% paraformaldehyde and permeabilized by phosphate-buffered saline with Tween 20 (PBST) (0.2% Triton X-100) for 30 min at room temperature (RT). After rinsing thrice for 5 min in phosphate-buffered saline (PBS), endplate chondrocytes were incubated in a blocking solution containing 1% bovine serum albumin, 5% goat serum, and 0.1% Triton X-100 in PBS for 2 h prior to the incubation with light chain 3 II (LC3II) antibody (1: 500, #ab222776, Abcam, Cambridge, UK) at 4°C overnight. Following washing six times for 10 min with PBST (0.1% Triton X-100), endplate chondrocytes were incubated with fluorescein isothiocyanate conjugated goat anti-rabbit IgG (H + L) (1: 200, # GB22303, Servicebio, Wuhan, Hubei, China) for 1 h at RT. Next, endplate chondrocytes were washed six times for 15 min in PBST and then photographed using a fluorescence microscope (DMI6000B, Leica, Wetzlar, Germany).

### 2.6 Western blot (WB)

Total proteins were extracted from mouse endplate chondrocytes by radioimmunoprecipitation assay buffer (#9806, Cell Signaling Technology, Danvers, MA, USA). Then, proteins were separated using sodium dodecyl sulfate-polyacrylamide gel electrophoresis and transferred onto a polyvinylidene fluoride membrane. Subsequently, membranes were blocked with 5% nonfat milk for 1 h at RT followed by the incubation with the indicated primary antibody overnight at 4°C. The next day membranes were washed with Tris-buffered saline containing 0.1% Tween 20 and incubated with the corresponding secondary antibody at RT for 1 h. Finally, signals of target proteins were identified by a chemiluminescence detection kit (#P0018F, Beyotime Biotechnology). The primary antibodies used for WB were matrix metallopeptidase (MMP) 3 antibody (1:1,000, #17873-1-AP, Proteintech, Wuhan, Hubei, China), MMP13 antibody (1:1,000, #18165-1-AP, Proteintech), LC3II antibody (1:1,000, #ab222776, Abcam, Cambridge, UK), Parkin antibody (1:1,000, #bs-23687R, Bioss), NAT10 antibody (1:2,000, #ab194297, Abcam), ESR1 antibody (1:1,000, #21244-1-AP, Proteintech), and β-actin (1:10,000, #bs-0061R, Bioss).

### 2.7 Acetylated RNA immunoprecipitation (acRIP)

Mouse endplate chondrocytes were first lysed to collect nucleic acid fragments which were then interrupted by ultrasound. Subsequently, cell lysate was incubated with ac4C antibody (1:500, #ab252215, Abcam) at 4°C overnight to enrich acetylated RNA fragments. Next, ac4C antibody combined with acetylated RNA fragments were captured using avidin magnetic beads, and the level of acetylated PINK1 mRNA was detected by qRT-PCR.

### 2.8 Dual-luciferase reporter gene assay

First, the wildtype (WT) *NAT10* gene promoter was cloned into a luciferase reporter gene vector (pGL3). Then, we mutated the ESR1 binding sites in the WT *NAT10* gene promoter to construct the luciferase reporter gene vector containing the mutated *NAT10* gene promoter (MUT). Next, mouse endplate chondrocytes were co-transfected with the WT or MUT *NAT10* gene promoter-luciferase reporter gene vector and siRNA against ESR1. Subsequently, endplate chondrocytes were collected at 48 h post-transfection to measure the luciferase activity using Dual-Luciferase Reporter Assay System (#E1910, Promega, Madison, WI, USA).

### 2.9 Statistical analyses

In the present study, quantitative data were presented as mean ± standard error of the mean (SEM), and statistical analyses were performed using SPSS (version 21.0, IBM Corp., Armonk, NY, USA). Comparisons of continuous variables between two groups were analyzed using the Student’s *t* test, while statistical differences among three or more groups were performed by the Kruskal–Wallis test. Besides, all statistical tests were two-sided, and *P* values <0.05 were considered statistically significant.

## 3 Results

### 3.1 STM attenuates endplate chondrocyte degeneration

**Figure 1:**
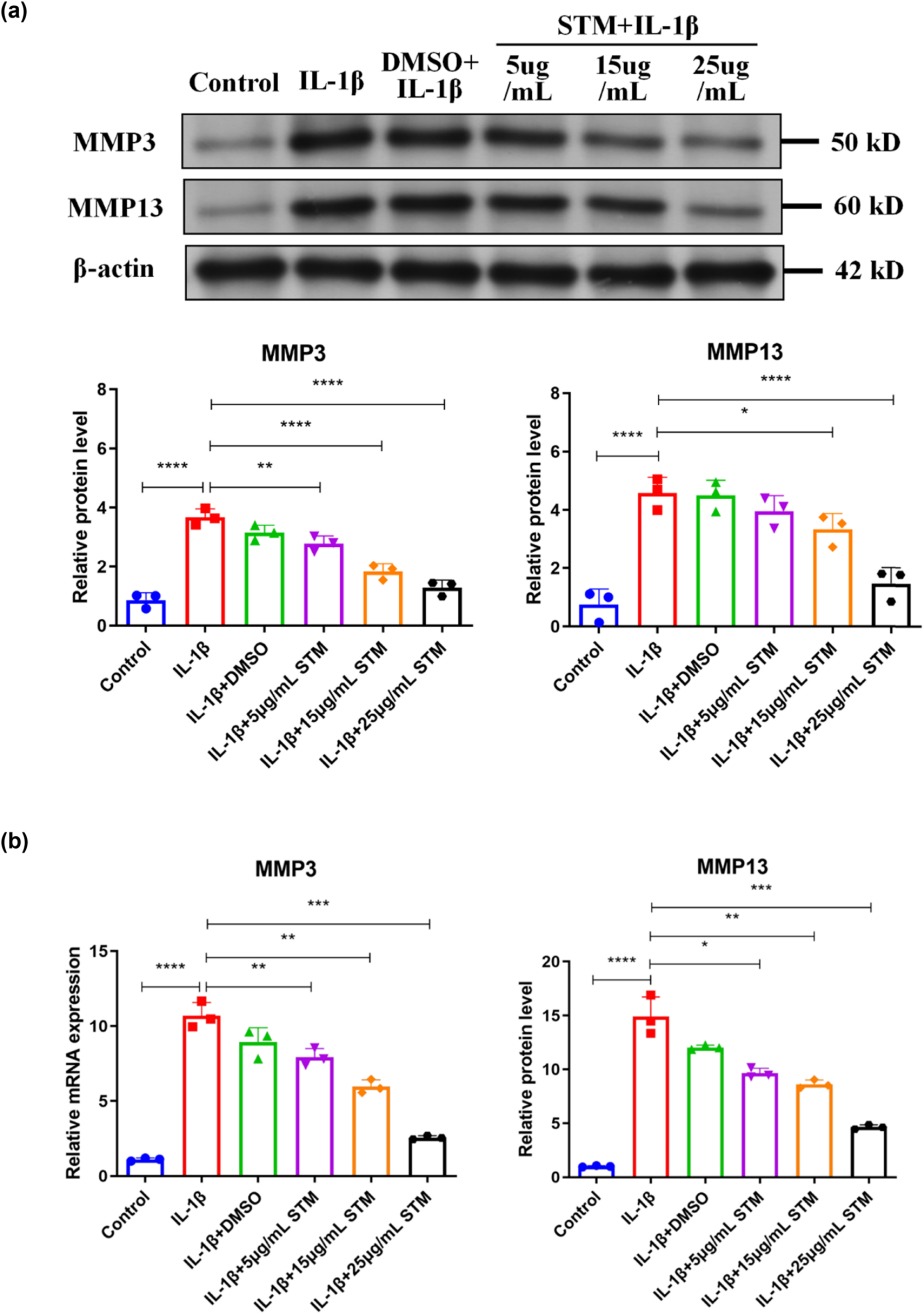
STM alleviates the degeneration of endplate chondrocytes. (a) Protein levels of MMP3 and MMP13 in mouse endplate chondrocytes treated with or without IL-1β or IL-1β plus STM. (b) mRNA levels of MMP3 and MMP13 in mouse endplate chondrocytes treated with or without IL-1β or IL-1β plus STM. STM: stigmasterol. *P < 0.05, **P < 0.01, ***P < 0.001, ****P < 0.0001.

To induce degeneration *in vitro*, mouse endplate chondrocytes were treated with IL-1β. Results indicated that IL-1β treatment increased protein and mRNA levels of degeneration markers, including MMP3 and MMP13, in endplate chondrocytes (Figure 1a and b). However, STM treatment declined IL-1β-induced protein and mRNA levels of MMP3 and MMP13 in a dose-dependent manner in endplate chondrocytes (Figure 1a and b). Besides, DMSO had no effect on MMP3 and MMP13 levels in endplate chondrocytes (Figure 1a and b). These data suggested that STM could alleviate endplate chondrocyte degeneration.

### 3.2 STM induces PINK1-mediated mitophagy in degenerated endplate chondrocytes

**Figure 2:**
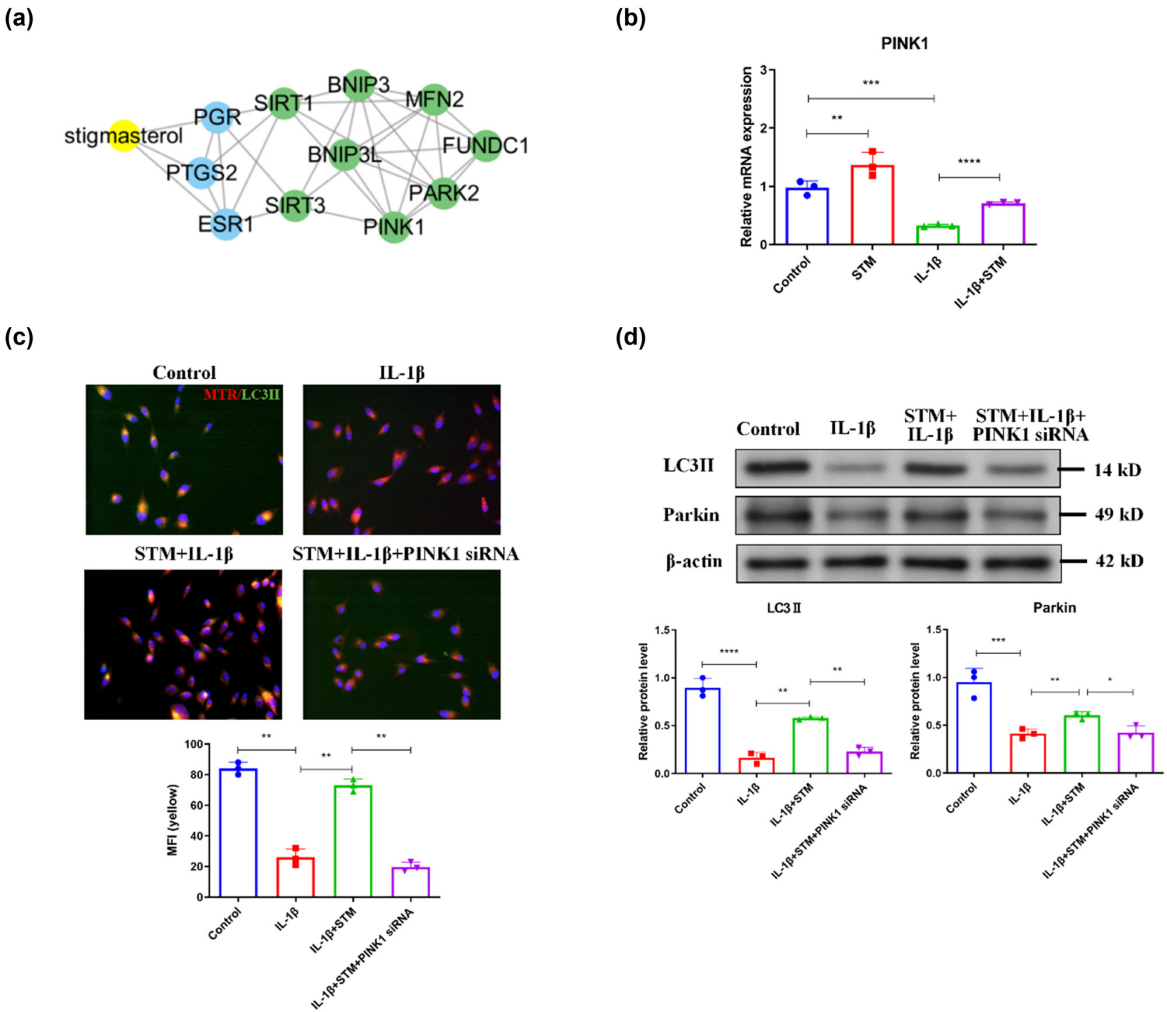
STM triggers PINK1-mediated mitophagy in degenerated endplate chondrocytes. (a) A network constructed based on the HERB database to analyze the correlation between STM and genes involved in mitophagy. (b) The mRNA level of PINK1 in mouse endplate chondrocytes treated with or without IL-1β or IL-1β plus STM. (c) The co-location of MTR and LC3II in mouse endplate chondrocytes treated with or without IL-1β or IL-1β plus STM. (d) Protein levels of LC3II and Parkin in mouse endplate chondrocytes treated with or without IL-1β or IL-1β plus STM. STM: stigmasterol; MTR: MitoTracker Red. *P < 0.05, **P < 0.01, ***P < 0.001, ****P < 0.0001.

As mitophagy could attenuate IVDD through hindering the degeneration of endplate chondrocytes by preventing mitochondrial dysfunction [12,13], the effect of STM on mitophagy in degenerated endplate chondrocytes was identified. Then, a network was constructed based on the HERB database to analyze the correlation between STM and genes involved in mitophagy. It was indicated that STM should be related to PINK1, a critical factor for mitophagy [18,19] (Figure 2a). Further qRT-PCR analysis showed that IL-1β treatment decreased PINK1 expression, whereas STM treatment abolished the inhibitory effect of IL-1β treatment on PINK1 expression in endplate chondrocytes (Figure 2b). Besides, STM also increased PINK1 expression in control endplate chondrocytes (Figure 2b).

Next, endplate chondrocytes were stained by mitochondrial marker MTR and autophagy marker LC3II via IF. Results of IF revealed that IL-1β treatment decreased the co-location of MTR and LC3II, while STM treatment enhanced the co-location of MTR and LC3II in endplate chondrocytes treated with IL-1β (Figure 2c). However, the silence of PINK1 by siRNA (Figure S1a) diminished the effect of STM treatment on the co-location of MTR and LC3II in endplate chondrocytes treated with IL-1β (Figure 2c). Similarly, WB results demonstrated that IL-1β treatment reduced the levels of mitophagy marker Parkin and LC3II, while STM treatment increased the levels of Parkin and LC3II in endplate chondrocytes treated with IL-1β (Figure 2d). Moreover, PINK1 silence neutralized the effect of STM treatment on the levels of Parkin and LC3II in endplate chondrocytes treated with IL-1β (Figure 2d). The above results suggested that STM could induce PINK1-mediated mitophagy in degenerated endplate chondrocytes.

### 3.3 NAT10 increases PINK1 expression by RNA acetylation in endplate chondrocytes

**Figure 3:**
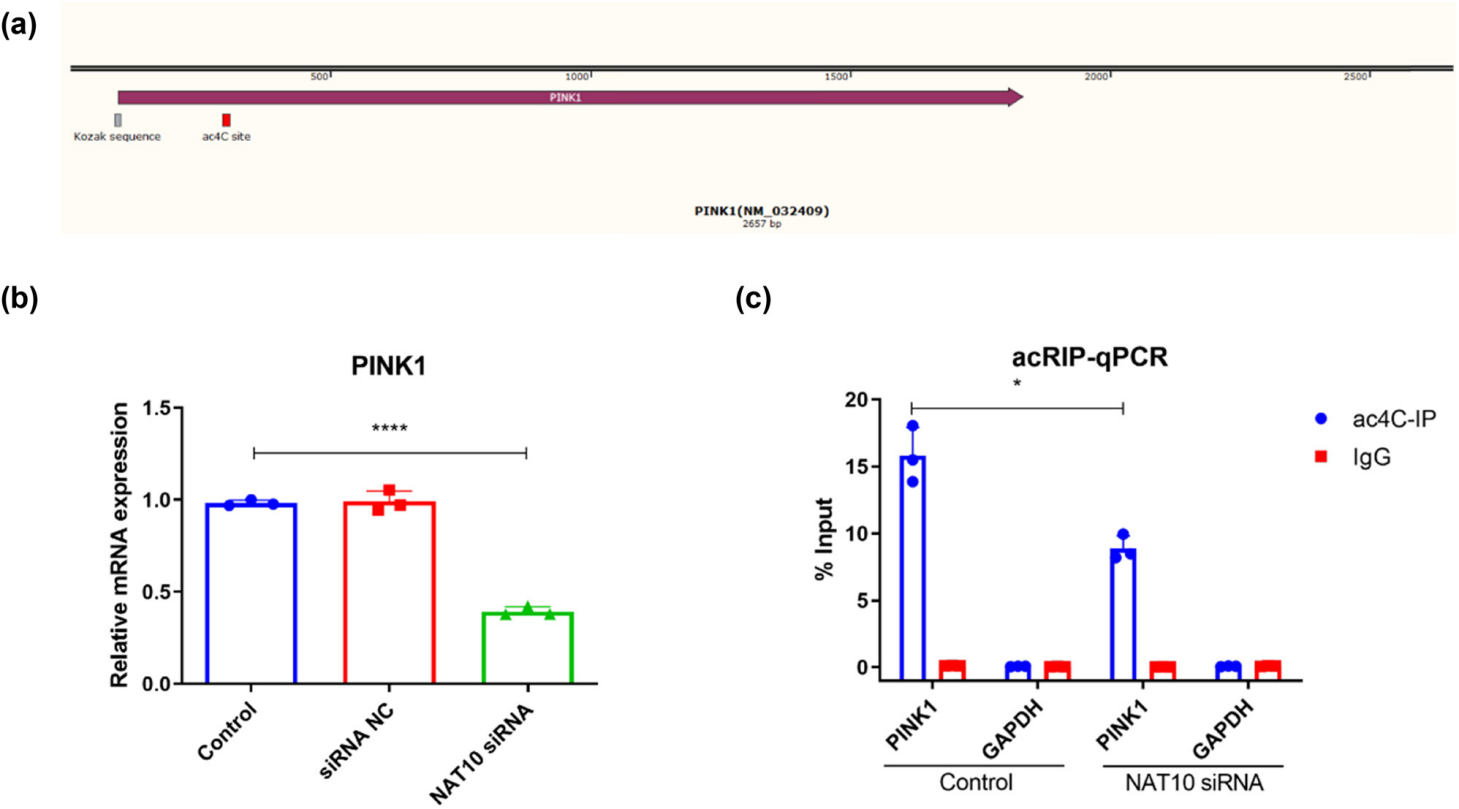
NAT10 elevates PINK1 expression by regulating RNA acetylation in endplate chondrocytes. (a) The potential ac4C site in PINK1 mRNA. (b) The mRNA level of PINK1 in mouse endplate chondrocytes treated with or without NAT10 siRNA. (c) The ac4C level of PINK1 mRNA detected by acRIP in mouse endplate chondrocytes treated with or without NAT10 siRNA. NC: negative control. *P < 0.05, ****P < 0.0001.

In addition, the mechanism of how STM increased PINK1 expression in endplate chondrocytes was explored. Bioinformatics analysis found a potential ac4C site in PINK1 mRNA (Figure 3a). Besides, the silence of NAT10 by siRNA (Figure S1b) declined the PINK1 mRNA level, while siRNA NC had no effect on the PINK1 mRNA level in endplate chondrocytes (Figure 3b). Moreover, acRIP results indicated that NAT10 silence reduced the PINK1 mRNA acetylation level (Figure 3c). All these results suggested that NAT10 increased PINK1 expression by improving PINK1 mRNA acetylation in endplate chondrocytes.

### 3.4 STM regulates NAT10 expression by ESR1 in degenerated endplate chondrocytes

**Figure 4:**
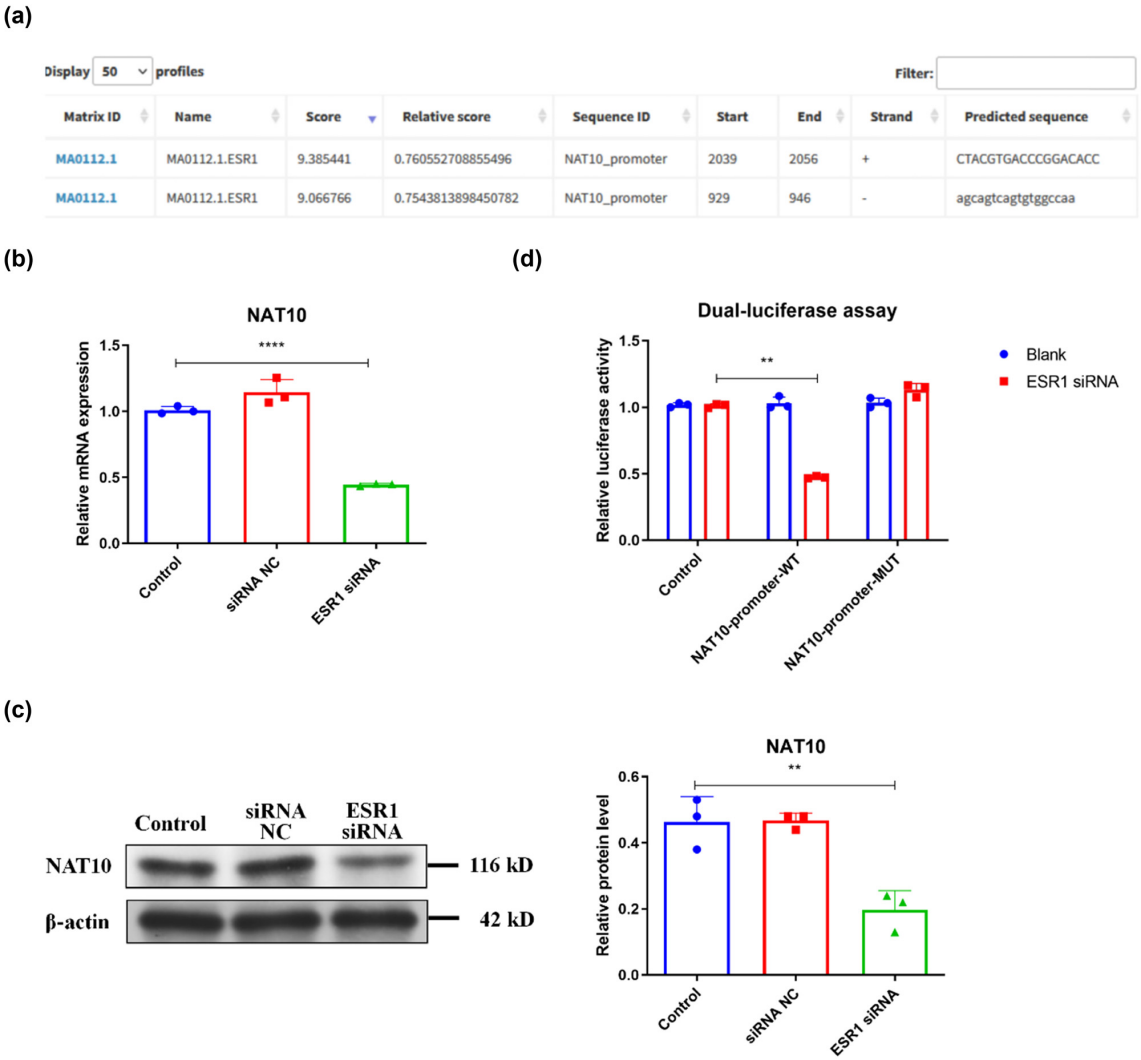
ESR1 promotes *NAT10* gene transcription in endplate chondrocytes. (a) The potential ESR1 binding sites in *NAT10* gene promoter. (b) The mRNA level of NAT10 in mouse endplate chondrocytes treated with or without ESR1 siRNA. (c) The protein level of NAT10 in mouse endplate chondrocytes treated with or without ESR1 siRNA. (d) The transcription activity of WT or MUT *NAT10* gene promoter was analyzed by relative luciferase reporter activity assay in mouse endplate chondrocytes treated with or without ESR1 siRNA. NC: negative control. **P < 0.01, ****P < 0.0001.

Next, the mechanism modifying NAT10 expression in degenerated endplate chondrocytes was further determined. Bioinformatics analysis using the JASPAR database found potential ESR1 binding sites in the *NAT10* gene promoter (Figure 4a). Besides, qRT-PCR results showed that silence of ESR1 by siRNA (Figure S1c) decreased NAT10 mRNA and protein levels, but siRNA NC had no effect on NAT10 mRNA and protein levels in endplate chondrocytes (Figure 4b and c). Moreover, a dual-luciferase reporter gene assay revealed that ESR1 silence reduced WT *NAT10* gene promoter activity, whereas it had no effect on that of the MUT *NAT10* gene promoter containing mutated ESR1 binding sites (Figure 4d). These results suggested that ESR1 could enhance the transcription of the *NAT10* gene in endplate chondrocytes.

**Figure 5:**
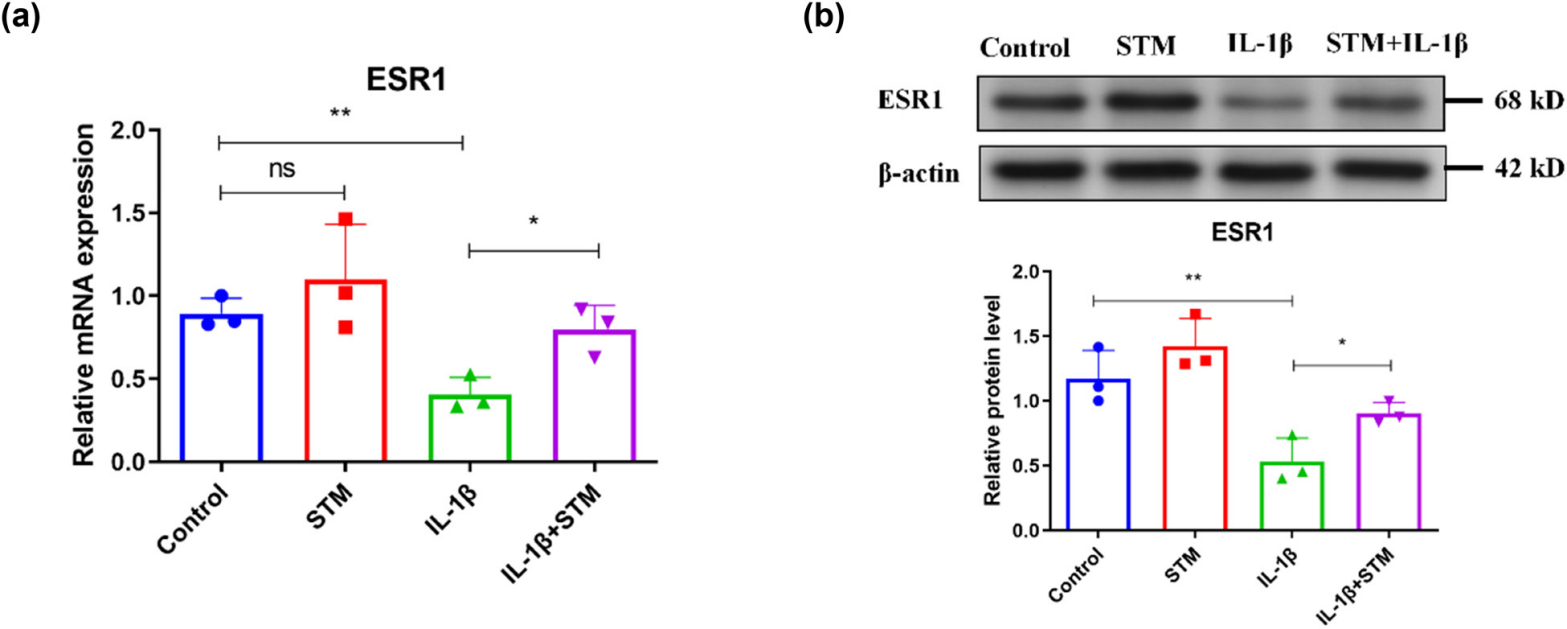
STM increases NAT10 expression by ESR1 in degenerated endplate chondrocytes. (a) The mRNA level of ESR1 in mouse endplate chondrocytes treated with or without IL-1β or IL-1β plus STM. (b) ESR1 protein level in mouse endplate chondrocytes treated with or without IL-1β or IL-1β plus STM. STM: stigmasterol. *P < 0.05, **P < 0.01.

The network constructed to analyze the correlation between STM and genes involved in mitophagy indicated that ESR1 should be associated with STM (Figure 2a). Therefore, the effect of STM on ESR1 was investigated. Results of qRT-PCR indicated that IL-1β treatment decreased the ESR1 mRNA level, while STM treatment restored the IL-1β-reduced ESR1 mRNA level in endplate chondrocytes (Figure 5a). Besides, STM also increased the ESR1 mRNA level in control endplate chondrocytes (Figure 5a). Similarly, WB results found that IL-1β treatment reduced the ESR1 protein level, whereas STM treatment increased the ESR1 protein level in endplate chondrocytes treated with IL-1β (Figure 5b). Moreover, STM treatment neutralized the effect of IL-1β treatment on the ESR1 protein level in endplate chondrocytes (Figure 5b). In addition, STM also increased the ESR1 protein level in control endplate chondrocytes (Figure 5b). All these data together suggested that STM regulated NAT10 expression by ESR1 in degenerated endplate chondrocytes.

## 4 Discussion

**Figure 6:**
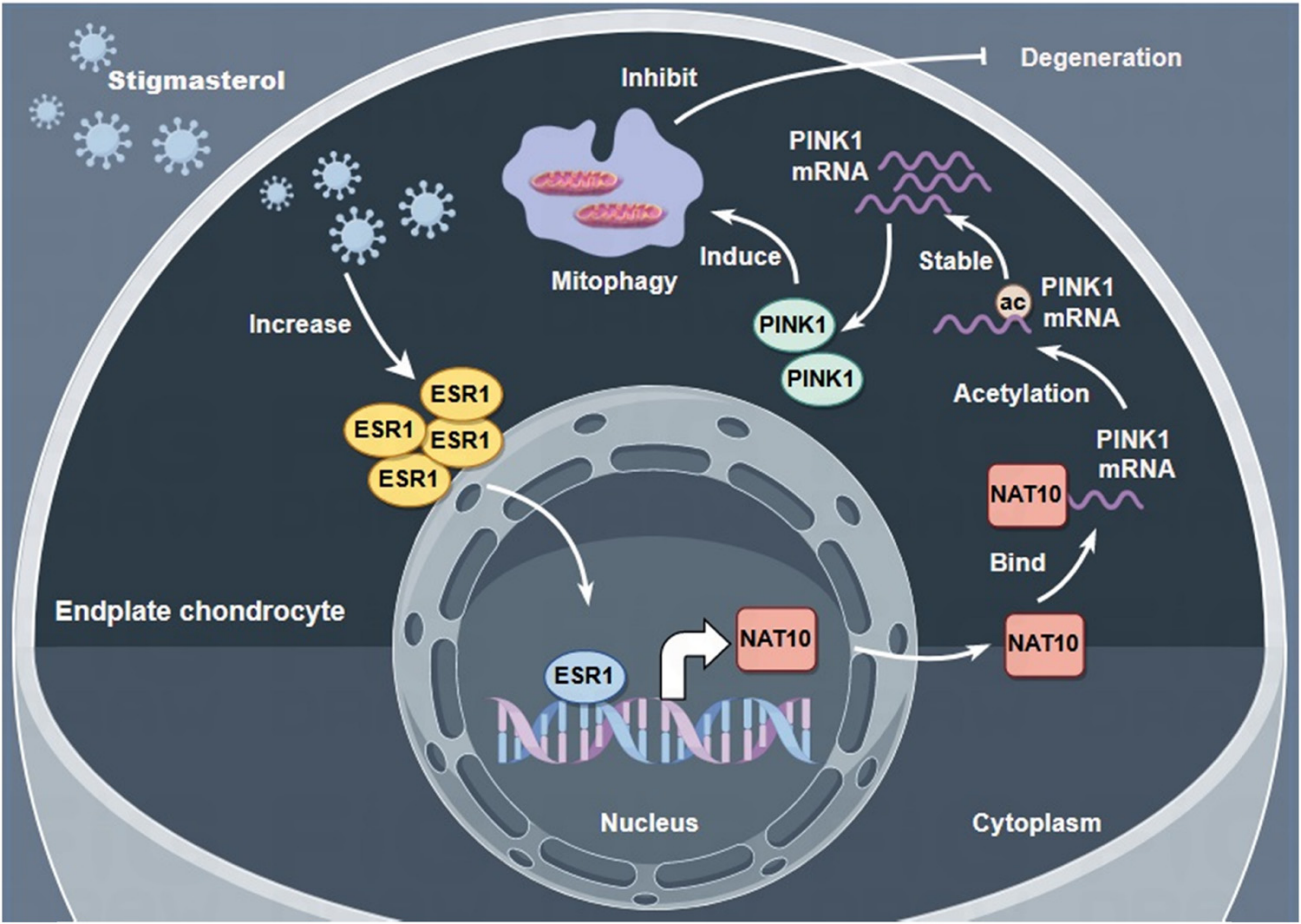
Schematic diagram of regulatory mechanisms for this study. This study revealed that STM alleviated endplate chondrocyte degeneration. Besides, STM induced PINK1-mediated mitophagy in degenerated endplate chondrocytes. Moreover, NAT10 increased PINK1 expression by improving PINK1 mRNA acetylation in endplate chondrocytes. In addition, STM regulated NAT10 expression by ESR1 in degenerated endplate chondrocytes.

This study revealed that STM alleviated endplate chondrocyte degeneration. Besides, STM induced PINK1-mediated mitophagy in degenerated endplate chondrocytes. Moreover, NAT10 increased PINK1 expression by improving PINK1 mRNA acetylation in endplate chondrocytes. In addition, STM regulated NAT10 expression by ESR1 in degenerated endplate chondrocytes (Figure 6).

Several studies have revealed the protective role of STM in chondrocytes. For instance, STM attenuates IL-1β-induced injuries by suppressing ferroptosis in mouse chondrocytes ATDC5 cells [20]. Besides, STM alleviates injuries of rat articular chondrocytes in osteoarthritis by negating IL-1β-induced inflammation [21]. However, the effect of STM on endplate chondrocytes in IVDD has not been reported. Thus, the current study uncovered the effect of STM on endplate chondrocytes in IVDD for the first time.

To date, only two studies have indicated the effect of STM on mitophagy [14,15]. Nevertheless, previous studies have not investigated the mechanism of STM regulating mitophagy. This study revealed that STM induced mitophagy by increasing PINK1 expression via improving PINK1 mRNA acetylation in endplate chondrocytes. Growing evidence has uncovered that ac4C RNA acetylation regulates RNA expression [17,22]. However, the role of ac4C RNA acetylation in PINK1 mRNA expression has not been reported. Therefore, our findings indicated that STM induced mitophagy by enhancing PINK1 mRNA acetylation in endplate chondrocytes for the first time.

This study found that NAT10 elevated the PINK1 mRNA level by enhancing PINK1 mRNA acetylation in endplate chondrocytes. NAT10-mediated acetylation could increase RNA levels by improving its stability. For example, NAT10 increases the notch receptor 3 (NOTCH3) mRNA level by improving mRNA stability via acetylation in cancers [23]. Besides, NAT10 stabilizes AHNAK nucleoprotein (AHNAK) mRNA to increase the AHNAK mRNA level by protecting it against exonucleases [24]. Therefore, NAT10 should increase the PINK1 mRNA level by improving its stability via acetylation in endplate chondrocytes. In addition, the role of NAT10-mediated RNA acetylation in mitophagy has not been investigated, which was explored for the first time in the present study.

A previous study has demonstrated that ESR1 could induce mitophagy by suppressing *DNA damage inducible transcript 3 (DDIT3/CHOP)* and *OMA1 zinc metallopeptidase (OMA1)* transcription as a transcription factor in pancreatic β-cells [25]. By contrast, ESR1 might induce mitophagy in mouse muscle [26]. Our findings also indicated that ESR1 should enhance *NAT10* transcription to trigger mitophagy in degenerated endplate chondrocytes, the first to uncover the regulatory role of ESR1 in NAT10. All these studies together suggest that ESR1 should exert opposite effects on mitophagy in different cells.

However, there were still several limitations of this study. For example, the current study could be strengthened by determining the functional relevance of RNA acetylation and NAT10 to mitophagy and degeneration of endplate chondrocytes. Besides, the findings of this study should be further confirmed by *in vivo* experiments.

## 5 Conclusion

In summary, the current study indicated that STM attenuated endplate chondrocyte degeneration through inducing mitophagy by enhancing PINK1 mRNA acetylation via the ESR1/NAT10 axis. These findings would provide novel strategies for the treatment of IVDD.

## Supplementary Material

Supplementary Figure
