# Supplementary material for: Stigmasterol alleviates endplate chondrocyte degeneration through inducing mitophagy by enhancing PINK1 mRNA acetylation via the ESR1/NAT10 axis
Source: Open Life Sci. 2025 Apr 8;20(1):20220913. doi: 10.1515/biol-2022-0913 (PMC11992624; doi:10.1515/biol-2022-0913)
Supplement: Supplementary Figure [file biol-2022-0913-sm.pdf]

Supplementary material

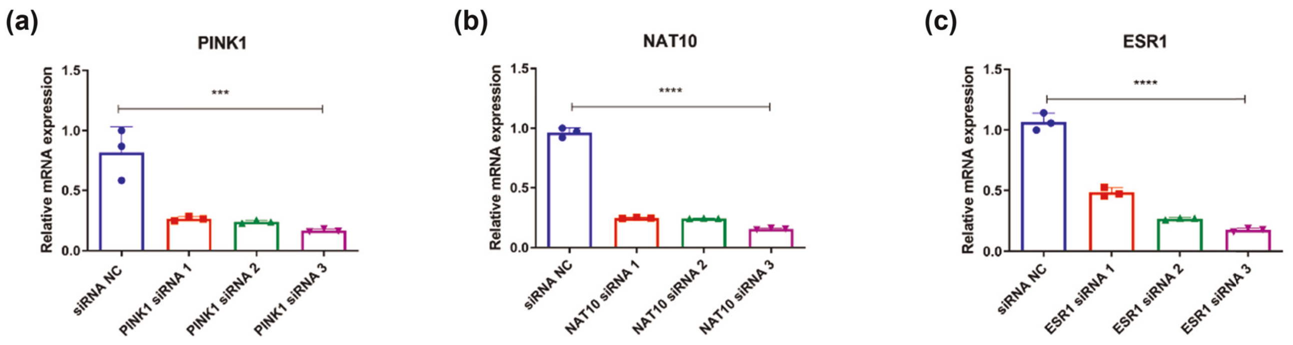

Figure S1: Efficiencies of siRNAs. The mRNA levels of PINK1 (a), NAT10 (b) and ESR1 (c) detected by qRT-PCR in MC3T3-E1 cells treated with or without siRNAs. NC, negative control. ***$P < 0.001$, ****$P < 0.0001$.
